# The Impact of Childhood Obesity on Adult Dental Caries: A Mendelian Randomization Study

**DOI:** 10.3390/dj13120573

**Published:** 2025-12-03

**Authors:** Yeon Woo Kim, Junhua Wu, Sun Woo Lim, Seongjin Lim, Su-Jeong Choi, Tae-Kyeong Lee, Sung Chul Choi, Dong Woon Kim

**Affiliations:** 1 Department of Oral Anatomy & Developmental Biology, Kyung Hee University College of Dentistry, Seoul 02447, Republic of Korea; nowywkim@khu.ac.kr (Y.W.K.); sunzy4498@khu.ac.kr (S.W.L.); seongjinlim0@gmail.com (S.L.); sujng486@gmail.com (S.-J.C.); tankado92@gmail.com (T.-K.L.); 2 Department of Neurology, Southwest Hospital, Third Military Medical University (Army Medical University), Chongqing 400038, China; key1030@126.com; 3 Department of Pediatric Dentistry, Kyung Hee University College of Dentistry, Seoul 02447, Republic of Korea; pedochoi@gmail.com

**Keywords:** SNPs, metabolic syndrome, cavity, childhood, mendelian randomization

## Abstract

**Background/Objectives:** This study investigates the association between childhood obesity and the incidence of dental caries in adulthood. The primary objective is to determine whether metabolic disorders experienced during the developmental years can have long-term impacts on oral health, thereby contributing to a more comprehensive understanding of dental growth and development. **Methods:** The research was conducted using single nucleotide polymorphisms (SNPs) from European populations within a Mendelian Randomization (MR) framework. **Results:** The analysis revealed a significant positive association between childhood obesity, BMI, and dental caries types 1 and 3, with OR values consistently higher than 1.05 or 1.12 (*p* < 0.05), except for MR Egger. ICD10 obesity was also strongly associated with dental caries 1 and 3. No significant associations were found between obesity/BMI and dental caries type 2, or between waist-to-hip ratio (adjusted for BMI) and dental caries (*p* > 0.05). **Conclusions:** The findings suggest that metabolic disorders during the growth period may have a lasting influence on the development of dental caries later in life. This research is the first study to explore the impact of childhood metabolic disorders on adult dental caries, highlighting its importance in the field of dental and public health.

## 1. Introduction

Dental caries, also known as tooth decay, is one of the most prevalent chronic diseases globally, affecting individuals throughout their lifetime. Caries occur when the vulnerable hard tissues of the teeth are locally destroyed by acidic byproducts produced from the bacterial fermentation of carbohydrates found in food [1,2]. It is a multifactorial disease initiated by microbiological shifts within a complex biofilm and influenced by factors such as saliva flow and composition, fluoride exposure, dietary sugar intake, and preventive behaviors like tooth brushing [3]. Dental caries can be classified into Class 1, 2, and 3 based on American Dental Association (ADA). In the United States, it is the most common chronic childhood disease, occurring five times more frequently than asthma. The incidence of dental caries is also rising among the elderly in the U.S. and other regions, as more individuals retain their teeth throughout their lives [4]. And in this study, childhood caries refers to caries in primary teeth, and adult caries refers to caries in permanent teeth.

Since the turn of the 21st century, childhood obesity rates have steadily increased. According to recent reports from the World Health Organization (WHO) and numerous large-scale epidemiological studies, the prevalence of obesity in children and adolescents (ages 5–19) is increasing significantly worldwide. The WHO reports that by 2022, approximately 1.6 million children and adolescents will be obese, and more than 3.9 million will be overweight or obese. A large-scale meta-analysis published in 2024 estimated the prevalence of obesity in children and adolescents aged 0–18 to be 8.5% (95% CI 8.2–8.8), indicating a steady increase, although this prevalence varies by region and economic status.

According to the China National Survey on Students’ Constitution and Health, the prevalence of obesity among children and adolescents aged 7–18 has surged from 0.1% in 1985 to 7.3% in 2014 [5,6]. WHO announced that obesity prevalence among children in U.S, aged 6–11 increased from 11.3% in 1988–1994 to 19.6% in 2007–2008. Studies have shown that childhood obesity contributes to various adult diseases, including coronary heart disease, myocardial infarction, heart failure, and atrial fibrillation, indicating that its effects extend beyond childhood into adult health [7].

Childhood obesity primarily results from an imbalance between energy intake and expenditure, heavily influenced by environmental factors, lifestyle choices, and cultural context. While genetics play a role in determining obesity risk, they account for less than 5% of cases. Factors like physical activity, family meal structure, and parental feeding styles also contribute. Socio-cultural influences, such as the use of food as a reward or social tool, further exacerbate unhealthy eating habits and sedentary behavior, leading to increased obesity rates in children [8]. The relationship between obesity and dental caries varies by age, with differences likely influenced by age-related factors, socioeconomic status, and research methods. Frequent sugar intake is a significant contributor to both conditions, and its impact can vary depending on the child’s age [9]. Particularly in obese adolescents, frequent sugar intake can lead to a threefold increase in caries incidence compared to their normal-weight peers, highlighting the need for targeted interventions in this population [10]. And there’s also biological rationale linking, as obesity-induced metabolic disorders are closely related to dental health [11]. In particular, poor oral hygiene associated with a high-calorie/high-sugar diet has been identified as a major pathway that increases the risk of both obesity and dental caries. Against this backdrop, this study aimed to elucidate the causal effect of obesity on dental caries based on recent data.

The relationship between obesity and oral health has been extensively investigated, with particular attention given to the association between obesity and dental caries [12]. Numerous studies have examined this connection in adults, revealing a notable correlation [13,14], although the findings remain a subject of ongoing debate. However, many researchers have proved this, showing that overweight children exhibit a higher incidence of caries [15,16,17]. These findings underscore the importance of addressing both obesity and dental health in children through targeted interventions. Despite these insights, there is limited genome-wide association study (GWAS) analysis on the extension of childhood obesity and dental caries into adulthood, indicating an area for future research.

This study tests the hypothesis that childhood obesity causally influences the development of dental caries in adulthood. In other words, we aim to identify a causal pathway through which childhood metabolic disease status exerts long-term effects on dental health using Mendelian randomization (MR) analysis. However, these studies might be influenced by various risk factors such as health and oral care status, possibly leading to reverse causality. Mendelian randomization (MR) is a method used in epidemiology to determine causality by using genetic predictors as instrumental variables. This technique helps reduce the impact of common confounders like environmental and socioeconomic factors, as well as individual behaviors [18,19].

MR is a representative instrumental variable-based analysis method. It utilizes genetic variants (SNPs) for causal inference. Since genetic variants are randomly inherited from parents, they can avoid the confounding influences of environment and lifestyle habits, as well as the problem of reverse causality. Therefore, we evaluated the causal effect of specific metabolic exposures on the development of dental caries in adulthood using MR (two-sample MR) analysis. MR supports the validity and innovation of this study design as a powerful methodology that can relatively clearly elucidate the causality between exposure and outcome even in multifactorial diseases with complex pathophysiology [20].

To overcome the limitations of previous research, we applied a two-sample MR analysis in this study to investigate the potential causal link between childhood obesity and dental caries. This study examines the permanent lasting impact of childhood obesity on oral health in adulthood, a topic of recent global concern.

The hypothesis of this study is that metabolic abnormalities experienced in childhood, particularly obesity, causally influence the development of adult dental caries. The primary goal of this study is to infer the causal relationship between childhood obesity and adult dental caries using genetic instrumental variables. Furthermore, we aim to evaluate the differences in causal effects across dental caries classes and to verify the robustness of our results by applying various MR protocols.

## 2. Materials and Methods

### 2.1. Study Design and Data Source

Study design Two-sample MR is considered a method of identifying the causal relationship between the phenotype of exposure and the outcome by using genetic variants for exposure as instrument variables (IV), which could make use of the accessible public dataset from larges ample genome-wide association studies (GWAS) for both “exposures” (as a risk factor) and “outcomes” (as a disease) and make up for typical shortcomings of observational studies.

This study is a secondary data review of existing databases. This study was designed based on the following three assumptions: (1) The Relevance Assumption: that the chosen independent variables (IVs) are directly associated with the exposure of interest; (2) The Independence Assumption: that the chosen IVs are not associated with any confounder variables between the exposure and outcome; (3) The Exclusion Restriction assumption: the chosen IVs do not affect the outcome, except through their association with the exposure [21,22]. The two-sample MR analysis was used to assess the causal association of childhood obesity (exposure) and Childhood BMI as Supplementary Data S1 (exposure) with the risk of Dental caries sub-type 1,2,3 (outcome).

In this case, we classify dental caries into three stages based on the primary area of the tooth affected. Dental caries progresses through the structural layers of the tooth, manifesting distinctly in the enamel, dentin, and dentin including little pulp. The classification of dental caries in this study was based on the ICD classification of dental caries. When describing the content in the dataset used for dental caries, the clinical situation was not clearly described, and it was classified into mild, moderate, and severe.

This study is focused on understanding the relationship between childhood obesity and dental caries. In the Dental caries 1, it is confined to the enamel, characterized by demineralization that presents as white spots without physical cavitation; the enamel surface remains smooth. In Dental caries 2, the caries are located in DEJ, where the softer dentinal tissue started to allow for rapid spread. This phase typically involves the formation of actual cavities, along with increased tooth sensitivity and discomfort. In the Dental caries 3, the carious lesion extends almost to the pulp, affecting the nerves and blood vessels housed within. Pulpal involvement leads to severe pain, and in advanced cases, pulp necrosis or abscess formation may occur, necessitating endodontic therapy. In extreme cases, tooth extraction is required.

Data source publicly available GWAS databases were searched to obtain eligible datasets of exposure and outcomes, including GWAS catalog, Neale lab, IEU open GWAS, and FinnGen. As such, no additional ethical approvals were required. Considering that the confounding of the population can lead to biased estimates, we limited the genetic background of the population for the MR study to individuals of European descent. The data provided by these organisations are compiled from surveys of large numbers of people. The data is open-sourced and anonymised to make it useful for large-scale, non-contact SNP studies such as MR, which is the method used in this study. The data on childhood obesity were obtained from a genome-wide association meta-analysis (GWAS ID: ieu-a-1096) conducted by the Early Growth Genetics (EGG) consortium. Here, the age range for childhood obesity is 1–19 years old. In the dataset labeled ieu-a-1096, a total of 5530 cases and 8318 controls were included, yielding a sample size of 13,848 participants. The analysis incorporated 2,442,739 SNPs, with the results expressed in log odds units [23].

Outcome data about dental caries were obtained from a publicly available GWAS dataset finngen, and classified into three subtypes (finngen_R11_K11_CARIES_1_OPER_ONLYAVO, finngen_R11_K11_CARIES_2_OPER_ONLYAVO, finngen_R11_K11_CARIES_3_OPER_ONLYAVO). This dataset originates from the Finngen Biobank, which includes genetic data from 174,108 individuals (102 case and 174,006 control) as of 2023, and also include meta-analyses of biobank data from Estonia and the United Kingdom [24]. finngen_R11_K11_CARIES_1_OPER_ONLYAVO, finngen_R11_K11_CARIES_2_OPER_ONLYAVO, and finngen_R11_K11_CARIES_3_OPER_ONLYAVO are all datasets extracted from the FinnGen database based on ICD (International Classification of Diseases) codes. These datasets contain dental caries (cavities) patients and their related dental surgery records (including outpatient avohilmo). However, these three datasets each reflect the progression of dental caries (mild → moderate → severe. Accordingly, CARIES_1 is likely to primarily represent the early (mild) stage of caries, CARIES_2 represents the moderate stage, such as dentin invasion, and CARIES_3 represents the severe stage, such as pulp invasion.

To assist with childhood obesity data, we also test childhood body mass index (BMI) with dental caries. And to compare data pattern with childhood obesity, Adult obesity, Body Mass Index (BMI), waist to Hip ratio adjusted to BMI was used as exposure. The data on childhood BMI were obtained from a genome-wide association meta-analysis (GWAS ID: ebi-a-GCST90002409) conducted by the EGG consortium. Sample size of 39,620 participants. The analysis incorporated 8,173,382 SNPs [25]. On this data, children are defined as those between the ages of 2–10. ICD 10: E66.9 Obesity were obtained from a genome-wide association meta-analysis (GWAS ID: ukb-b-15541) conducted by the UK Biobank consortium. In the dataset labeled ukb-b-15541, a total of 4688 cases and 458,322 controls were included, yielding a sample size of 463,010 participants. The analysis incorporated 9,851,867 SNPs, with the results expressed in SD units [26]. BMI Data were obtained from a genome-wide association meta-analysis (GWAS ID: ukb-a-248) conducted by the Neale Lab consortium. The Data are yielding a sample size of 336,107 participants. The analysis incorporated 10,894,596 SNPs, with the results expressed in SD units [27]. The data on Waist-to-hip ratio adjusted for BMI were obtained from a genome-wide association meta-analysis (GWAS ID: ebi-a-GCST90025996) conducted by the UK Biobank, yielding a sample size of 458,349 participants. The analysis incorporated 4,238,887 SNPs [28].

### 2.2. Selection of Genetic Instruments

In this study, SNPs that were independent of each other (R^2^ < 0.001) and strongly associated with the exposure factor (*p* < 5 × 10^−8^) were initially selected [29]. Potential confounders associated with the outcomes were identified and excluded using the PhenoScanner database V2, after which SNPs with palindromic and incompatible alleles were removed [30].

However, for childhood obesity, the number of SNPs meeting the *p*-value threshold of 5 × 10^−8^ was only 6, which was deemed insufficient for statistical power. To address this, a more lenient significance threshold (*p* < 1 × 10^−5^) was applied to ensure adequate statistical power (greater than 10 indicates a lower probability of weak instrument bias).

### 2.3. Statistical Analysis

Summary statistics of childhood obesity and Dental caries have harmonized in terms of effect allele, and subsequent analyses were based on the merged exposure-outcome dataset. Statistical analysis This two-sample MR analysis was performed using R software (version 4.1.2, R Foundation for Statistical Computing, Vienna, Austria) with TwoSampleMR (version 0.5.6) and MR-PRESSO packages (version 1.0.0).

In this study, we employed Mendelian Randomization (MR) as a robust method to investigate the causal relationship between the exposure and outcome variables. The primary analytical approach utilized was the Inverse Variance Weighted (IVW) model, supplemented by four additional methods: Weighted Median Estimator (WME), Weighted Model-Based (WM), MR-Egger, and MR-Robust Modified Profile Score (MRAPS).

The IVW model, serving as the principal analytical tool, efficiently combines the causal estimates associated with each single nucleotide polymorphism (SNP) by applying weights inversely proportional to their variance. This approach allows for a relatively stable and accurate causal estimation by using a meta-analytic method to combine Wald estimates across instrumental variables (IVs) when directional pleiotropy is absent. However, the IVW model can be sensitive to potential biases arising from pleiotropic effects, given its assumption of no horizontal pleiotropy [31,32].

To mitigate these limitations, we incorporated the WME approach, which is capable of providing reliable causal estimates even when up to 50% of the genetic instruments are invalid. This method ensures the robustness of our findings, particularly in the presence of pleiotropy, by reducing Type I error and offering a more accurate assessment of causal relationships under these conditions [33]. WM method was applied, which constructs the optimal model by assigning weights to the genetic instruments. This approach is particularly advantageous in scenarios where the genetic architecture of the exposure is complex or not fully understood, offering greater flexibility in handling invalid instruments. Furthermore, the MR-Egger regression model was utilized to adjust for pleiotropy. Although MR-Egger may suffer from reduced statistical power, it provides an unbiased estimate of causal effects by controlling for directional pleiotropy through the regression slope and intercept [34,35,36].

The F-statistic plays a crucial role in Mendelian randomization (MR) studies. The F-statistic is a numerical value that assesses the strength of the association between a genetic instrumental variable and an exposure variable (e.g., a disease or behavioral factor), allowing us to determine whether the instrumental variable has a sufficiently strong influence on the exposure. A high F-value (typically 10 or greater) reduces the risk of weak instrumental bias and increases the reliability of MR analysis results. Conversely, a low F-value can indicate instability or bias in the analysis results, making it crucial to clearly recognize and report limitations when interpreting the results. Therefore, each F-statistic has been calculated and attached as a separate file.

### 2.4. Sensitivity and Validation Analysis

We employed the MRAPS model to address residual confounding, offering particularly robust estimates even in the presence of pleiotropy and other confounding factors. This method is highly effective in ensuring the reliability of causal inference across a wide range of research contexts. By utilizing these diverse MR analytical approaches, our study was able to derive reliable causal inferences while accounting for the complex genetic and environmental interactions between the exposure and outcome [37]. This comprehensive approach helps to address the potential limitations of each method, thereby enhancing the overall robustness and reliability of our findings.

To demonstrate the validity of positive control outcomes, we conducted an analysis using “Diagnoses—secondary ICD10: I10 Essential (primary) hypertension” (GWAS ID: ukb-b-12493) as the exposure and “Stroke” (FinnGen R11, ID: finngen_R11_C_STROKE) as the outcome [38,39]. The purpose of this analysis was to establish the increased likelihood of stroke incidence associated with hypertension. The findings (Supplementary Data S1) confirmed that individuals diagnosed with essential hypertension have a significantly higher risk of developing stroke, thereby validating the methodology employed in this study.

## 3. Results

### 3.1. Childhood Obesity and Dental Caries

**Figure 1:**
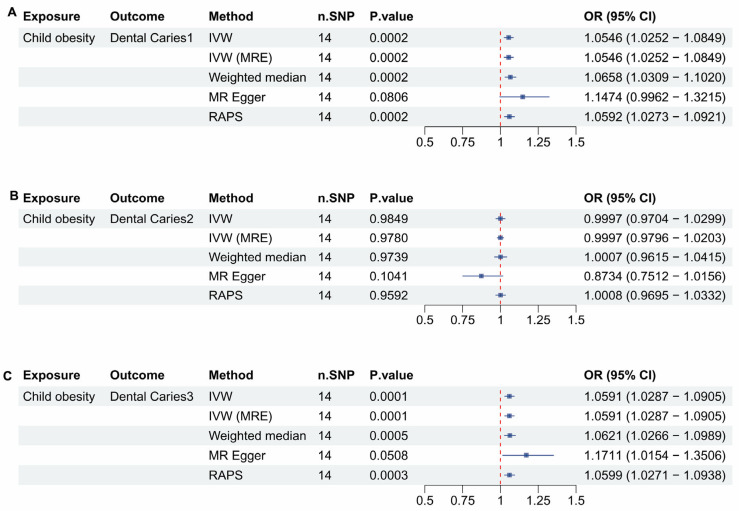
Mendelian Randomization (MR) analyses between childhood obesity and dental caries types 1, 2, and 3. (**A**) forest plot showing the MR analysis results for Child Obesity and Dental Caries 1, (**B**) forest plot showing the MR analysis results for Child Obesity and Dental Caries 2, (**C**) forest plot showing the MR analysis results for Child Obesity and Dental Caries 3.

There are 14 SNPs included in the Childhood Obesity dataset. In Figure 1A, the analysis of childhood obesity and Dental Caries 1 using the Inverse Variance Weighted (IVW) method revealed a significant positive association, with a *p*-value of 0.002, an OR of 1.00546, and a 95% confidence interval (CI) of 1.0252 to 1.0849. All methods except MR Egger indicated OR bigger than 1.05, so significant positive correlation (*p* < 0.05). In Figure 1B, the analysis showed no significant association between childhood obesity and Dental Caries 2 (*p* > 0.05). However, the analysis of childhood obesity and Dental Caries 3 also demonstrated a significant positive association using the IVW method, with a *p*-value of 0.002, an OR of 1.00546, and a 95% CI of 1.0252 to 1.0849 in Figure 1C. Similarly to the findings in Figure 1A, all methods except MR Egger confirmed a significant positive correlation, because their OR is bigger than 1.05 (*p* < 0.05) (Table 1).

### 3.2. Childhood BMI and Dental Caries

**Figure 2:**
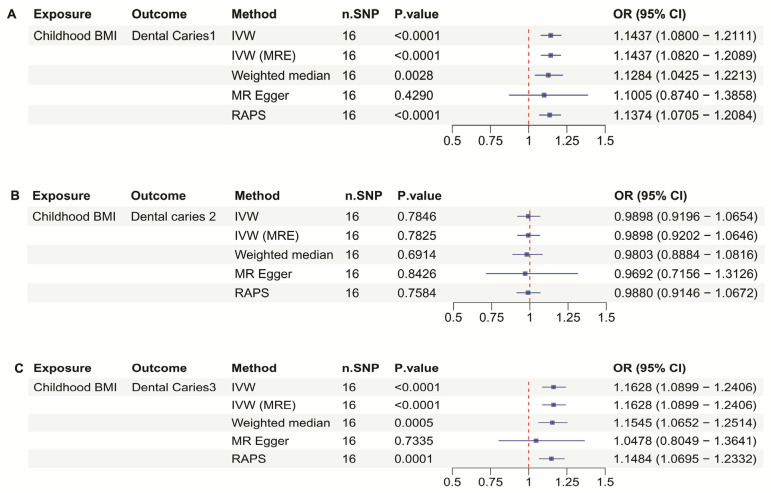
Mendelian Randomization (MR) analyses between Childhood Body Mass Index and dental caries types 1, 2, and 3. (**A**) forest plot showing the MR analysis results for Childhood Body Mass Index (BMI) and Dental Caries 1, (**B**) forest plot showing the MR analysis results for Childhood Body Mass Index and Dental Caries 2, (**C**) forest plot showing the MR analysis results for Childhood Body Mass Index and Dental Caries 3.

There are 16 SNPs included in the Childhood BMI dataset. In Figure 2A, the analysis of Childhood BMI and Dental Caries 1 revealed a highly significant association using the Inverse Variance Weighted (IVW) method, with a *p*-value < 0.0001, an OR of 1.1437, and a 95% CI of 1.0800 to 1.2111 indicating a strong positive correlation. All other methods, except for MR Egger, also demonstrated a significant and strong positive correlation (*p* < 0.05). And they have OR bigger than 1.12. In Figure 2B, the analysis indicated no significant association between childhood BMI and Dental Caries 2 (*p* > 0.05). The analysis of childhood BMI and Dental Caries 3 again showed a significant association using the IVW method, with a *p*-value < 0.0001, an OR of 1.1628, and a 95% CI of 1.0899 to 1.2406 confirming a strong positive correlation in Figure 2C. Similarly to the findings in Figure 2A, all other methods, except MR Egger, also indicated a significant positive correlation (OR bigger than 1.14 (*p* < 0.05)) (Table 2).

### 3.3. ICD10 Obesity and Dental Caries

**Figure 3:**
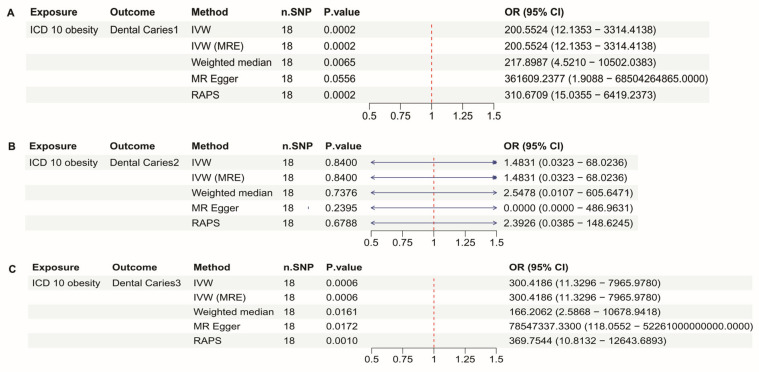
Mendelian Randomization (MR) analyses between ICD 10 Obesity and Dental caries types 1, 2, and 3. (**A**) forest plot showing the MR analysis results for ICD 10 Obesity and Dental Caries 1, (**B**) forest plot showing the MR analysis results for ICD 10 Obesity and Dental Caries 2, (**C**) forest plot showing the MR analysis results for ICD 10 Obesity and Dental Caries 3.

There are 18 SNPs included in the ICD10 obesity dataset. In Figure 3A, the association between ICD 10 Obesity and Dental Caries 1 was analyzed. The Inverse Variance Weighted (IVW) method demonstrated a significant association with a *p*-value of 0.0002, an OR of 200.5524, and a 95% CI ranging from 12.1353 to 3314.4138, indicating a strong positive correlation. The MR Egger method also suggested a weak association with a *p*-value of 0.0556, and all other methods confirmed a strong positive correlation which all OR is bigger than 200 (*p* < 0.056). In Figure 3B, the analysis showed no significant association between obesity and Dental Caries 2, as reflected by a *p*-value greater than 0.05. In Figure 3C, significant association using the IVW method, with a *p*-value < 0.0001, an OR of 1.1628, and a 95% CI of 1.0899 to 1.2406, confirming a strong positive correlation in Figure 1C. All other methods also demonstrated significant and strong positive correlations (*p* < 0.05), and their ORs are larger than 166.

### 3.4. BMI and Dental Caries

**Figure 4:**
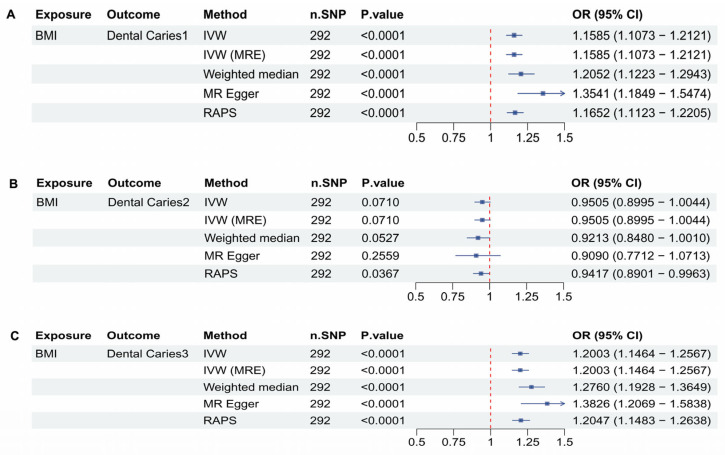
Mendelian Randomization (MR) analyses between BMI and Dental caries types 1, 2, and 3. (**A**) forest plot showing the MR analysis results for BMI and Dental Caries 1, (**B**) forest plot showing the MR analysis results for BMI and Dental Caries 2, (**C**) forest plot showing the MR analysis results for BMI and Dental Caries 3.

There are 292 SNPs included in the BMI dataset. In Figure 4A, the association between BMI and Dental Caries 1 was analyzed. The Inverse Variance Weighted (IVW) method revealed a strongly significant positive association, with a *p*-value < 0.0001, an OR of 1.1585, and a 95% CI ranging from 1.1073 to 1.2121. Other methods also demonstrated strong significance and positive correlations, OR bigger than 1.15 (*p* < 0.0001). In Figure 4B, the analysis showed no significant association between BMI and Dental Caries 2, as indicated by a *p*-value > 0.05. In Figure 4C, the analysis of BMI and Dental Caries 3 revealed a very strongly significant positive association using the IVW method, with a *p*-value < 0.0001, an OR of 1.2003, and a 95% CI of 1.1464 to 1.2567. All other methods also confirmed significant and strong positive correlations (*p* < 0.05), and the OR is bigger than 1.2.

### 3.5. Waist to Hip Ratio Adjusted to BMI and Dental Caries

**Figure 5:**
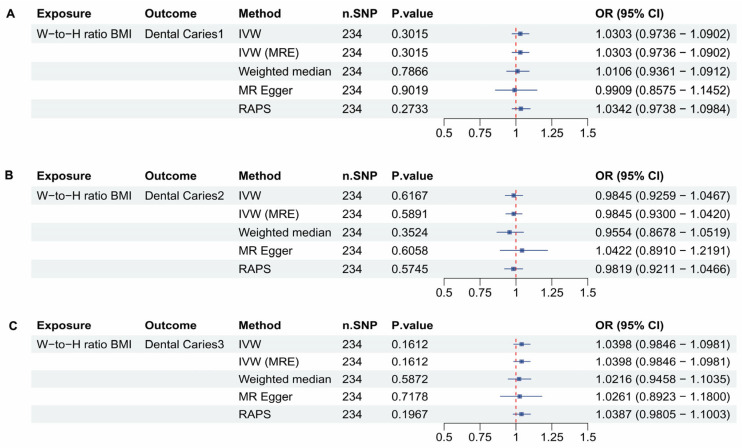
Mendelian Randomization (MR) analyses between Waist to Hip ratio adjusted to BMI and Dental caries types 1, 2, and 3. (**A**) forest plot showing the MR analysis results for Waist to Hip ratio adjusted to BMI and Dental Caries 1, (**B**) forest plot showing the MR analysis results for Waist to Hip ratio adjusted to BMI and Dental Caries 2, (**C**) forest plot showing the MR analysis results for Waist to Hip ratio adjusted to BMI and Dental Caries 3.

There are 234 SNPs included in the Waist to Hip ratio adjusted to BMI dataset. In Figure 5A–C, the analysis yielded non-significant results (*p* > 0.05).

## 4. Discussion

This study suggests a potential causal relationship between childhood obesity and the incidence of dental caries in adulthood.

Figure 1 demonstrates that childhood obesity is significantly correlated with dental caries affecting both enamel and deep dentin almost including pulp. When compared with the results in Figure 2, a similar trend is observed, which helps to compensate for the lowered *p*-value threshold used in the childhood obesity data. However, the odds ratio (OR) is considerably higher in Figure 2. Upon analyzing the differences, it was identified that the childhood obesity dataset (ieu-a-1096) encompasses individuals under the age of 18, while the childhood BMI dataset (ebi-a-GCST90002409) focuses on BMI in children aged 2 to 10 years.

This suggests that the impact of metabolic disorders experienced at a growth spurt age is greater on the development of dental caries. Notably, this period coincides with the formation and eruption of permanent teeth, typically between ages 6 to 12 [40]. There was a study by Anu and colleagues found a significant correlation between BMI and the eruption status of permanent teeth [41]. Through this, we can suggest that child obesity and BMI may influence the process of permanent tooth formation.

If we hypothesize the potential pathways through which childhood obesity could impact the process of tooth formation. Obesity, as a metabolic disorder, often leads to imbalances in various minerals and hormones within the body, including a potential deficiency in calcium—a critical element in the tooth formation process [42,43]. Increased insulin resistance due to childhood obesity can have a negative impact on the process of tooth formation. Insulin resistance affects not only glucose metabolism in the body but also various metabolic pathways, influencing the growth and development of tissues, particularly hard tissues like teeth. In obese children, insulin resistance may interfere with the formation of enamel, leading to weaker teeth or developmental abnormalities [44,45].

Interestingly, childhood obesity did not show a significant effect on DEJ, likely due to the continuous repair process of tertiary dentin. Dentin is regenerated through the differentiation and mineralization of odontoblasts and dental pulp stem cells (DPSCs), in which bioactive molecules such as TGF-β1 and the Wnt/β-catenin signaling pathway play crucial roles [46]. TGF-β1 promotes odontoblast differentiation and reparative dentinogenesis, while also regulating inflammatory responses to suppress the progression of metabolic disorders. Additionally, the Wnt/β-catenin pathway enhances cell survival and differentiation, contributing to resilience against metabolic stress. DPSCs aid in tissue regeneration and inflammation regulation, potentially recovering damage caused by metabolic diseases. Scaffold materials such as calcium silicate and bioceramics can further modulate inflammation within the pulp tissue and promote reparative dentinogenesis, thereby strengthening resistance against metabolic diseases. Therefore, it is hypothesized that the regenerative capacity of dentin in response to metabolic disorders may have influenced the observed outcomes.

Figure 3 and Figure 4 further highlight that adult obesity strongly influences the incidence of dental caries. However, However, the OR values for ICD- 10 obesity in Figure 3 show considerable heterogeneous. Although it is possible that obesity-related metabolic disorders have a substantial impact on dental health, an OR over 200 is unlikely to have a clear biological explanation. When revisiting the statistical structure of the dataset, the total sample size is 463,010 and n cases is 4688, meaning that obesity accounts for only about 1% of the population. As a result, the extremely high OR is likely influenced by rare-event bias, and the estimate may be inflated or distorted by residual external factors, especially for an outcome such as dental caries that is highly sensitive to environmental influences.

As shown in Figure 5, there was no significant association between the waist-to-hip ratio adjusted for BMI and dental caries, indicating that abdominal fat may not directly impact dental caries. This is an interesting finding that suggests the impact of BMI on different body parts is different. Further research is required to explore this aspect.

This study is the first to comprehensively evaluate the causal relationship between childhood obesity and adult dental caries, identifying 14 SNPs of child obesity and 16 SNPs of childhood BMI using GWAS datasets and analyzing the relationship through five different models. The analysis, after adjusting for potential outliers, confirmed a robust causal relationship between child obesity and dental caries, particularly affecting the enamel and pulp. These findings underscore the importance of lifelong oral health management for individuals with a genetic predisposition to child obesity. Multidisciplinary preventive interventions are recommended, including nutrition and hygiene education at school and community levels, family-centered lifestyle improvement programs, and early fluoride application and regular checkups at pediatric dental clinics. Furthermore, given that both obesity and caries share common risk factors, such as dietary habits and socioeconomic environment, this supports the need for integrated public health policies.

However, this study has some limitations. First, the MR Egger method yielded non-significant results, likely due to its lower statistical power compared to other Mendelian Randomization methods. This issue is particularly pronounced with smaller sample sizes or weaker instrumental variables [35,36].

And while the results of this analysis suggest a link between childhood obesity and adult dental caries, this does not necessarily mean that it is a direct causal effect. Alternative pathways through which genetic variants may influence obesity (e.g., other metabolic changes besides insulin resistance), residual multiple effects (pleiotropy), reverse causation and possibility, and behavioral mediators not reflected by genes (e.g., dietary habits, oral hygiene behaviors) may also be involved.

Additionally, the GWAS data for child obesity and child BMI used in this study had certain limitations. The SNPs did not meet the standard bioinformatic threshold of (*p* < 5 × $10^{-8})$ [47,48], leading us to adopt a less stringent significance level (*p* < 1 × 10^−5^). While this approach was necessary, it introduces the possibility of weak instrument bias, necessitating caution in interpreting the results.

Lastly, this study was conducted exclusively with European populations, which may limit the generalizability of the findings to other ethnic groups. Further research is needed to validate these results across diverse populations [49].

## 5. Conclusions

In conclusion, there is a causal relationship between childhood obesity and adult dental caries. This result indicates that individuals with a history of childhood obesity require attention to their oral heath to prevent dental caries. Further studies are needed to examine the biological mechanisms underlying this association.

## Figures and Tables

**Table 1:** Summary table between childhood obesity and dental caries type 1, 2, and 3.

Outcome in Childhood Obesity	IVW OR 95%Cl	IVW *p*-Value	MRE OR 95%Cl	MRE *p*-Value	WM OR 95%Cl	WM *p*-Value	MR Egger OR 95%Cl	MR Egger *p*-Value	RAPS OR 95%Cl	RAPS *p*-Value
Caries 1	1.0546 (1.0252–1.0849)	0.0002	1.0546 (1.0252–1.0849)	0.0002	1.0658 (1.0309–1.1020)	0.0002	1.1474 (0.9962–1.3215)	0.0806	1.0592 (1.0273–1.0921)	0.0002
Caries 2	Not valid	0.9849	Not valid	0.9780	Not valid	0.9739	Not valid	0.1041	Not valid	0.9592
Caries 3	1.0591 (1.0287–1.0905)	0.0001	1.0591 (1.0287–1.0905)	0.0001	1.0621 (1.0621–1.0989)	0.0005	Not valid	0.0508	1.0599 (1.0271–1.0938)	0.0003

**Table 2:** Summary table between childhood Body Mass Index and dental caries type 1, 2, and 3.

Outcome in Childhood BMI	IVW OR 95%Cl	IVW *p*-Value	MRE OR 95%Cl	MRE *p*-Value	WM OR 95%Cl	WM *p*-Value	MR Egger OR 95%Cl	MR egger *p*-Value	RAPS OR 95%Cl	RAPS *p*-Value
Caries 1	1.1437 (1.0800–1.2111)	<0.0001	1.1437 (1.0820–1.2089)	<0.0001	1.1284 (1.0425–1.2213)	0.0028	1.1005 (0.8740–1.3858)	0.4290	1.1374 (1.0705–1.2084)	<0.0001
Caries 2	Not Valid	0.7846	Not valid	0.7825	Not valid	0.6914	Not valid	0.8426	Not valid	0.7584
Caries 3	1.1628 (1.0899–1.2406)	<0.0001	1.1628 (1.0899–1.2406)	<0.0001	1.1545 (1.0652–1.2514)	0.0005	Not valid	0.7335	1.1484 (1.0695–1.2332)	0.0001

## Data Availability

The data presented in the study are available on request from the corresponding author. All bona fide researchers can apply for and access the public datasets from the UK Biobank (https://www.ukbiobank.ac.uk/, accessed on 23 June 2025), FinnGen project (https://www.finngen.fi/en, on 23 June 2025), and MEGASTROKE consortium (https://www.megastroke.org/, on 23 June 2025). The MRC-IEU (https://gwas.mrcieu.ac.uk/, on 23 June 2025) and FinnGen websites provide download links for all the data used in this study.

## Supplementary Materials

The following supporting information can be downloaded at: https://www.mdpi.com/article/10.3390/dj13120573/s1, Supplementary Data S1.
